# Transitioning from Medicaid to private health insurance: informing public and private sector outreach

**DOI:** 10.3389/frhs.2023.1166034

**Published:** 2023-09-01

**Authors:** Amy Samantha White

**Affiliations:** Department of Health Equity, Administration, and Technology, School of Health Sciences, Human Services, and Nursing, Lehman College, Bronx, NY, United States

**Keywords:** Medicaid, health insurance, transition, college graduate, copays

## Abstract

This study explored the lived experiences of transitioning from Medicaid to private health insurance upon college graduation. Fifteen recent graduates of an urban, commuter, public college in the Mid-Atlantic were interviewed via Zoom® to understand what they regard as crucial aspects of the transition experience, especially during the COVID pandemic. The subjects all identified as being members of a minority racial or ethnic group, the average age was 33 years (SD = 10.96), and all but one interview subject majored in the health sciences. Every recent graduate reported experiencing difficulty in the transition. Subjects felt unprepared for the transition, alone, and without support. “Copays” was the most common response to questions, frequently said with arms in the air for emphasis, as if the word “copay” summarized all of the lack of preparation, difficulty, and expense of the healthcare system after previously receiving Medicaid (i.e., free healthcare). The findings inform how the private sector should on-board new college graduates. There is a need for Medicaid case officers to better prepare clients for the transition and for human resources personnel in the private sector to sufficiently explain how private health insurance works.

## 1. Introduction

Medicaid enrollment grew during the COVID pandemic (1, 2). Enrollment increased both in states with and without Medicaid expansion (3). These increases were independent of the varying levels of economic shutdowns in the different states (4, 5) and can be attributed to the federal government giving incentives to states to keep Medicaid recipients enrolled during the pandemic, to layoffs that forced employees off their employer-sponsored health insurance and onto Medicaid, and to the need for healthcare during a global pandemic. This growth is limited. The current declining rates of COVID hospitalizations and deaths are resulting in these Medicaid disenrollment incentives being removed as of May 2023 (6). Researchers estimate that between 5 and 14 million people will lose their Medicaid insurance benefits (7). Some who are able will likely seek full-time employment from large employers to re-gain lost health insurance benefits.

This study targets a subset of those individuals seeking full-time employment: recent college graduates. Recent college graduates are of particular interest because of the data exploring the economic benefits of earning a bachelor's degree (8–12). Results from this study are useful for informing future research and making recommendations to private employers to promote innovation and minimize health inequities among their new hires who may be transitioning off Medicaid to private insurance for the first time. Results are also useful for Medicaid administrators to better prepare clients prior to disenrollment. An extant review of the current publications [Elton B. Stephens Company (EBSCO) and Google Scholar] did not identify any published research on transitioning from Medicaid to private insurance.

National survey data as well as local state-based surveys indicate that Medicaid recipients are satisfied with both their Medicaid insurance as well as the accessibility of physicians who accept Medicaid payment (13–16). Conversely, private health insurance satisfaction ranks fourth from the bottom in a comparison of consumer satisfaction, tied with the postal service and just above consumer satisfaction with their subscription television (17). Another national survey found that half of all private insurance recipients do not think their health plan demonstrated concern for their health after the COVID-19 pandemic stuck (18). James et al. (19), Norton et al. (20), and O'Connor and Kabadayi (21) studied health insurance literacy and how a lack of understanding and/or a lack of control negatively impact healthcare utilization, but none of these studies isolated individuals transitioning off Medicaid.

Discontinuing individuals’ Medicaid health benefits and requiring them to transition to private insurance, a form of health insurance with lower satisfaction ratings, raises two questions: (1) are these individuals prepared for the transition and (2) what would help them be successful in their transition from public to private health insurance? The purpose of this study was to describe (1) the lived experience of recent college graduates transitioning from Medicaid to private health insurance, (2) what the study participants regard as the crucial aspect(s) of their experience, and (3) how the private sector can minimize healthcare disruptions during these transitions.

## 2. Methods

In 2021, the author chose to use a phenomenological study design to build a body of knowledge by learning from the experience of recent college graduates. Phenomenology methods explore lived experiences by allowing subjects to describe their own experiences (22, 23). To develop processes to help individuals transition off Medicaid, the first step is to explore an individual's lived experiences through this transition. The research instrument included four, semi-structured, open-ended questions:

1. Tell me about your experience of having private health insurance for the first time.
2. How would you describe the transition from Medicaid to private health insurance?
3. What do you regard as the most helpful or important aspects of successfully transitioning off of Medicaid?
4. What else would you like to add about your experience transitioning from Medicaid to private health insurance?

A pilot study of three individuals led to the omission of the word “successful” to avoid confusion from individuals who did not deem their transition to be successful but could otherwise describe their experience transitioning health insurance payers.

### 2.1. Participant recruitment

Following Institutional Review Board approval from the A.T. Still University and criterion sampling, the alumni association of a public, urban, Hispanic-serving college emailed recent alumni (graduated within the last 2 years) who self-identified as having received Medicaid in college but now had private insurance. The email contained the study description and asked alumni who met the inclusion criteria to opt in by responding via email. The inclusion criteria included needing to have Medicaid health insurance while in college and, upon graduating, earned a salary that exceeded Medicaid's income threshold, and therefore the alum now had private health insurance. Email exchanges confirmed eligibility, and a Zoom interview was scheduled. Within the first week of the initial email invitation, a total of 30 individuals responded with 15 interviews conducted, two of which were later excluded from the final analysis for failure to meet all eligibility criteria.

### 2.2. Sample

There were 13 participants included in this study. All participants (100%) identified as a member of a racial or ethnic minority (i.e., Black, Latino/a, or Asian). Participants’ ages ranged from 23 to 56 years old, with an average age of 33 (standard deviation = 10.96). A majority (80%) of the study participants identified as female, and salaries ranged from a part-time salary of $25,000/year to $90,000/year.

### 2.3. Data collection

After respondents returned the informed consent, the author scheduled an online interview that was recorded in Zoom® and later transcribed. No respondents declined the recording or transcribing. On a case-by-case basis, the author used prompts such as “can you elaborate” or “tell me the ways” to obtain more detailed responses. The author also asked for interview participants to consider specific encounters, like an annual check-up, to compare their experience on Medicaid vs. private health insurance.

Interview responses were analyzed as they were collected to identify themes and patterns as well as to recognize when saturation was reached. Data saturation was reached when no new themes emerged, which was achieved after the 10th interview. However, the author proceeded with the five already scheduled interviews to gather more examples.

Interviews lasted approximately 45 min. Following the interview, the author conducted member checking by emailing study participants a summary of the response themes, highlighting if there were any ambiguities or issues for clarification. The author also sent the study participants a $20 Target e-gift card as a gesture of appreciation. Member checking resulted in confirmation of interview responses.

#### 2.3.1. Validity, reliability, and generalizability

Phenomenology is the appropriate research design to build a body of knowledge based on the experiences of those who have lived the event (24). Phenomenologies are subject-defined explorations of the lived experience by a defined group of people using their words, perceptions, and understanding (25). Phenomenological studies are not intended to be generalized from; phenomenological research has the smallest sample size of qualitative research. By Khan's (25) definition, a phenomenological research design ensures the validity of the research as the results are the lived experiences in their own words. The author ensured data reliability by consistently asking the same open-ended questions, in the same manner (26).

The author also ensured the validity of the research by rigorously following Morse's (27) six criteria for evaluating the qualitative research: credibility, confirmability, meaning in context, recurrent patterning, saturation, and transferability. The interviews were not intended to be representative of anyone except the individuals who experienced the transition. Demographic data were collected only to provide more context for participants’ lived experiences.

### 2.4. Data cleaning

The purpose of data cleaning was to clarify the intention of the interview subject and ensure that the data were an accurate reflection of the subjects’ experiences. Cleaning the interview transcripts (data) was a multi-step process culminating in coding the cleaned transcripts. Coding is not traditionally part of phenomenological data analysis (26). However, coding the data was helpful for organizing responses into themes. The final data coding procedure was an iterative process of reading and re-reading the transcripts following the steps of Giorgi et al. (28) for reviewing qualitative data.

## 3. Results

The overall themes that emerged from the interviews were that everyone who described the lived experience of transitioning from Medicaid to private insurance experienced felt unprepared, some degree of difficulty with the transition, and a sense of shock at the out-of-pocket expenses, most commonly expressed as “copays” said emphatically with hands in the air for emphasis. The words interview participants used to describe the transition were “difficult,” “shocking,” and “sudden,” leaving the interview participants scared and sometimes ashamed to ask for help.

**Figure 1:**
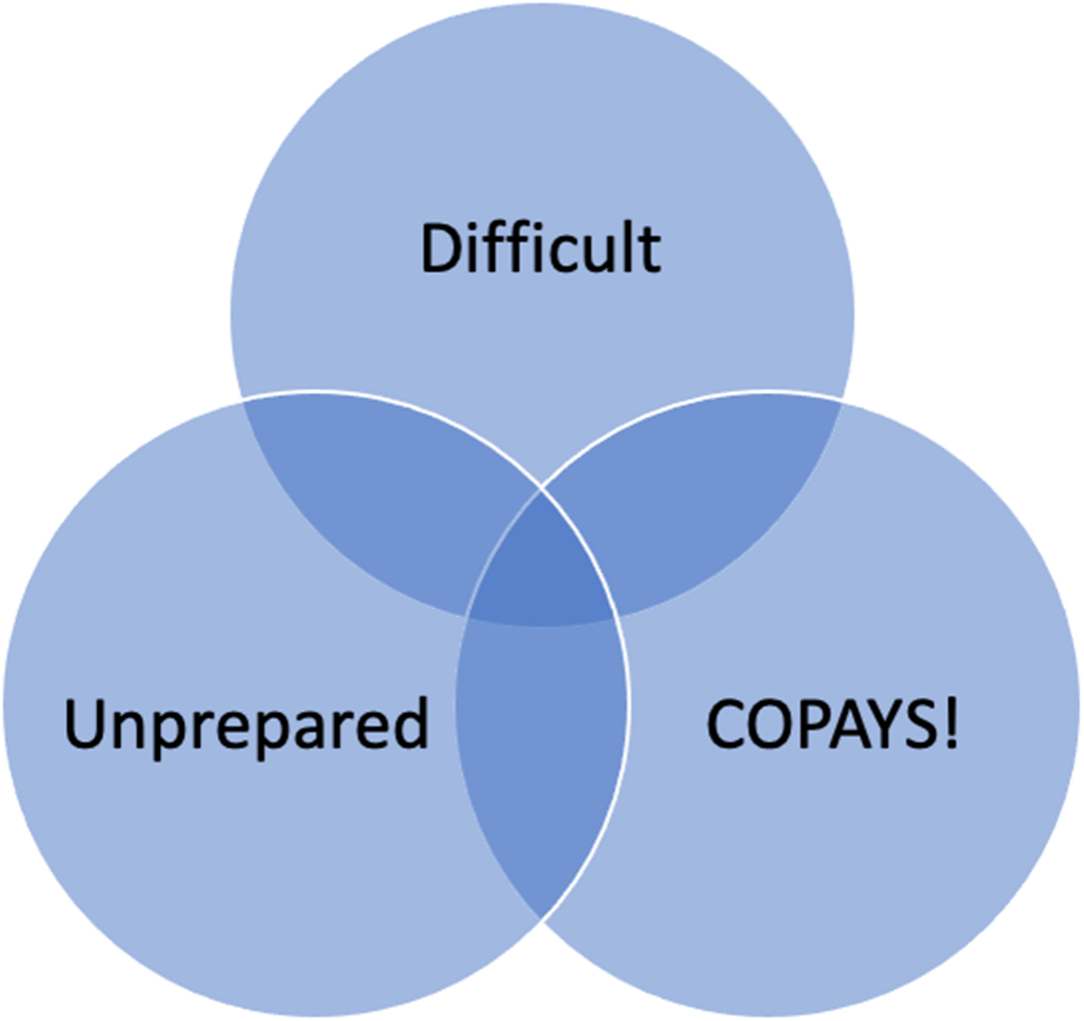
Venn diagram of overall study findings.

These main findings of overall themes are presented in Figure 1.

**Figure 2:**
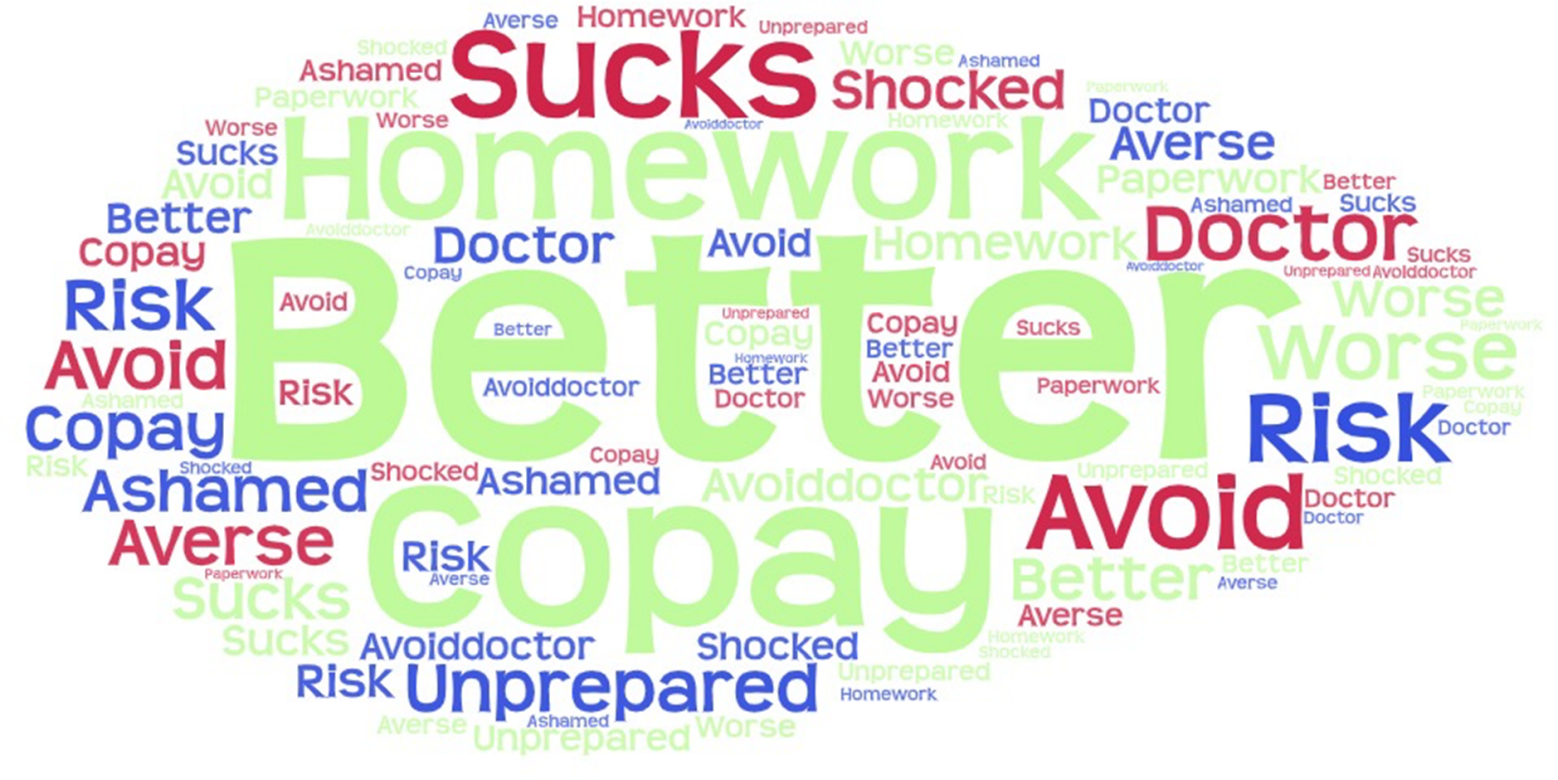
Word cloud of question 1 responses.

All but two of the interview subjects had prior awareness of the cost of private insurance or the cost of healthcare, yet all responded to interview questions by commenting on the cost of medical services. P2 said, “Medicaid did not prepare me for the expense of medical care.” P14 described the transition as, “You get something for free for a long time then you get a letter that your coverage will stop,” resulting in tremendous stress over the unknown costs ahead and a rush to use Medicaid benefits while coverage still existed. Every interview subject responded to questions about their transition by describing their lack of preparation. The most common responses in the interviews are presented in Figure 2. Transitioning from Medicaid—where patients see Medicaid-approved providers and there is no patient paperwork—to private insurance, where patients have to do their homework to identify who is in-network, and patients receive volumes of paperwork from their insurance providers, was an experience for which interview subjects felt unprepared and overwhelmed.

The study subjects had an awareness of improved access to providers with private insurance over Medicaid (62%), the sense of pride in this earned benefit (described by 46% of the interview sample), the feeling of being unsupported in the transition (46%), the feeling that they needed to change their behaviors to avoid copayments by not using their benefits (46%), and the feeling of being risk averse during a global pandemic (38%). The experience of improved access was summarized by P9, a parent of a child with special needs. P9 had previously relied on Medicaid's early intervention services and was unaware of the services available and covered by private insurance. P9 also emphasized the difference in wait times, appointment availability, and individualized attention at private facilities compared to the higher volume, Medicaid-accepting facilities. P11 similarly described her experience transitioning insurance types as finally having her choice of providers instead of being referred to one provider and not being offered a choice.

A similar theme through many of the interviews was a frustration and disappointment in the cost of medical care. Everyone mentioned “copays” as something they did not pay with Medicaid and that had now become the defining experience of receiving private medical insurance. P6 said, “I am afraid of copays” and does not use the private health insurance, preferring to use herbs and self-care. While P4 said that copays are so high, P4 believes health insurance is a scam because “doctors like copays too much.”

There are two distinct ways as presented in the data that individuals describe the lived experience of transitioning off Medicaid: a sense of pride and a sense of fear. The sense of pride even though private health insurance was expensive and often an expense the interview subject had no reference for. However, the study subjects felt a sense of pride at now being able to pay these costs on their own, not relying on the government or family, and appreciating the responsibility. The sense of fear came from a lack of preparation. Medicaid does not share invoices, bills, or any paperwork with recipients, so the interview subjects had neither context nor expectation of the premiums, copayments, deductibles, and/or coinsurance they faced.

Fear of not being able to pay for the high costs associated with private health insurance came from their lack of awareness or understanding of how private health insurance worked, and for some, their incredulity and distrust of the system. These interview subjects believed the system to be corrupt where “doctors like copayments”; these interview subjects did not use their health insurance benefits. Their understanding of the transition was from using their Medicaid whenever they felt it was medically appropriate to never using their private health insurance because they were afraid of the copayments. A summary of representative themed responses is presented in Table 1.

**Table 1:** Theme-to-text table.

Theme	Quote
Difficult	“I was ashamed of not understanding the insurance options.”
	“Losing Medicaid is like the rug getting pulled out from under you.” “Losing Medicaid felt like getting kicked when you’re already down.”
Copays	“Medicaid felt like a warm blanket there to cover me.”
Unprepared	“Nobody teaches you this in school.” “Parents don't teach their kids about this [health insurance].”
Improved access to providers	“Agencies that provide behavioral therapy (ABA) do not take Medicaid, only private insurance. If you have Medicaid, you don't even know that ABA exists, and that your kid isn't eligible for it.”
Unsupported	“Imagine somebody else did everything for you. For free. Then all of the sudden it's your turn? No support, no nothing.”
Change behaviors to avoid copayments	“I used to go to the dentist every six months. I can't remember the last time I went because I don't understand my new benefits.”
Risk averse	“I didn't have health insurance before; it was too expensive. But during open enrollment, I signed up. You can't not have health insurance during the pandemic.”
Sense of pride	“Not shocked. Not mad. It feels pretty awesome to do this.” “I’m not scared of the financial burden [of health insurance], I earned it.”

## 4. Discussion

The primary finding of the study was that 100% of the interview subjects experienced feeling unprepared for the transition. All respondents also described the steep learning curve associated with understanding premiums, copayments, deductibles, and finding in-network providers, all concepts that Medicaid recipients do not pay nor experience. Support services would similarly help Medicaid recipients’ transition to private health insurance and learn how to navigate the unfamiliar experience. These findings are in keeping with previous research by James et al. (19), Norton et al. (20), and O’Connor and Kabadayi (21) on how health insurance literacy and the lack of understanding of cost sharing and managed care impacts healthcare utilization.

The lived experience of paying for private health insurance after receiving free Medicaid varied. Approximately half of the interview subjects took pride in their new skillset and abilities to earn their families’ health benefits. There is a parallel between the finding of pride in the ability to earn one's own health benefits and the findings of Blakeslee and Best (29) and Munson et al. (30) who concluded that foster children who are successfully emancipated from the state are those who gain a sense of control over their circumstances. Taking pride in being able to pay for the healthcare that one once had to rely on the state to provide is a sense of control. This sense of control potentially contributed to the pride experienced by study subjects. These same interview subjects also responded to probes that they do not intend to ever be on Medicaid again.

The other half of interview subjects described feeling so uncertain and unfamiliar with the copayments, coinsurance, and deductibles that the interview subjects did not use their insurance, preferring to seek self-care and use over-the-counter treatments to avoid paying copayments. There are public health implications if insured individuals, like half of those interview subjects in this study, do not use their health insurance out of fear of paying an unknown copayment, coinsurance, or deductible or because they distrust the healthcare system. Research demonstrates how Medicaid recipients, by definition of their lower income, suffer a disproportionate amount of health issues relative to higher earners (31, 32). The consequences of former Medicaid recipients no longer seeking healthcare could have the same meaningful health effects.

All of the interview subjects experienced some degree of confusion upon transitioning to private health insurance. Payroll deductions, insurance vocabulary, and knowing who to ask for help were all topics of confusion. This research demonstrated that some individuals were too embarrassed to ask for help and felt alone in their transition. Recent college graduates from lower socio-economic backgrounds are often overwhelmed and fatigued by the experience of finishing their education, understanding their college debt, and transitioning to the working world, while still experiencing housing and food insecurity. Medicaid deemed the recent graduates as out-earning the income requirements; but to the graduates, they were only a few paychecks away from poverty with new expenses of college debt and health insurance costs. Understanding payroll deductions was not a common theme but is a topic worthy of follow-up. Also, interview subjects asked rhetorical questions about paying premiums for insurance while also sometimes having to pay copayments per encounter plus dental, lab, and vision benefits were not covered like other medical encounters, incurring coinsurance costs, a very different experience from Medicaid. There was confusion about what is purchased when a health insurance premium is paid, and why there are additional costs per service or encounter.

An unexpected finding from this research was that some Medicaid recipients were able to petition their Medicaid caseworker for an extension of not only Medicaid benefits but also food stamps for up to 90 days to help with their transition, while other Medicaid recipients were abruptly disenrolled without any prior warning. College students finishing their degree while living on poverty-level wages may have neither the support networks nor experience to know to ask for an extension of their benefits. As studied by Payne-Sturges et al. (33) and Broton and Goldrick-Rab (34), the impact of college students living in poverty results in food insecurity and impacts both physical and mental health, in addition to having consequences on academic performance. Recent college alumni could be given resource materials about their Medicaid benefits and how to maintain the benefits for a limited time while transitioning to private health insurance. Like P13 responded, annualized salaries may disqualify the individuals from Medicaid benefits. However, the individual is still living in poverty after receiving the first few paychecks. Paying a $25 copayment for a private insurance medical encounter is unaffordable if one has only earned a fraction of an annual salary.

Another common theme expressed during the interviews was that “you don't know what you don't know” (P1, P2, P5, P7, P10, P12, P13, and P14) until one has private insurance. In most states, Medicaid is a form of health insurance but with no paperwork and no cost to the recipient; the experience of having Medicaid is dissimilar from the experience of having private health insurance with the copious paperwork and costs. The individuals interviewed for this research study did not know that they were unprepared for the transition as they did not know the costs or paperwork involved with being a consumer of private health insurance. Furthermore, recipients of private health insurance have to research who is an in-network provider or pay the consequences of going out-of-network. Recipients of Medicaid rely on their Medicaid providers to make in-network referrals and the Medicaid recipients are not responsible for costs or research nor do they see any paperwork.

The findings from this study reveal an unmet need: former Medicaid recipients need educational materials and help selecting private health insurance and learning how to use their benefits. P4 described the emphasis in school on topics of professionalism. P4 felt well prepared to transition from school to the working world; however, none of the educational content on professionalism and how to manage work-related responsibilities helped with the specific skills and vocabulary needed to select a health insurance provider, read a paystub, identify in-network providers, and know how to use Healthcare savings accounts (HSA) money. P4 described experiencing a false sense of preparation because they had erroneously drawn an analogy between professionalism and real-world preparation. Similar to the first-generation college student support services and orientations, the author’s research demonstrates a need for support services as graduates transition from college to full-time employment. Many of the interview subjects do not have support networks and are the first in their family not to receive Medicaid.

## 5. Limitations

The limitations of this study were associated with problems caused by the COVID-19 pandemic that touched data collection, study population, and geographical location. Data collection occurred in January 2021 in the Mid-Atlantic region of the country at a time when COVID-19 diagnoses were at its post-holiday peak. There were limits on public transportation, restrictions on gatherings, and other laws to prevent spread of the highly transmissible virus. The impact of the global pandemic on individual's employment status and interest in participating in the study is unknown, but likely limited the ability to reach a larger population of potential study participants. The resulting small sample size limits generalizability. Similarly, only one state's Medicaid rules and communications are reflected, further limiting generalizability to larger populations.

The impacts of the global pandemic may have led to job losses or limitations on work hours, both leading to possible research participants remaining on Medicaid longer than they would have if the pandemic did not occur. It is also possible that study participants may not have been able to commit time and energy to participate in a study when the economic and health situation of the pandemic created more urgent needs and worries.

Another limitation of this research is that sampling mainly health-related majors may have impacted the findings of the study, in that only one of the interview subjects was from a non-health-related discipline (P4 was a computer science major). The interview subjects’ responses reflect their lived experiences of transitioning health insurance having studied health sciences while in college and now working in health-related fields (e.g., nursing, health administration, and social work). In addition, participants often use the word “copay” as an answer assuming the author understood the complexity of that answer, which then required the author to further question their experiences and intentions as to what they mean by “copay” explaining the health insurance transition.

## 6. Recommendations for future research

The first recommendation for future research is a larger version of this study conducted with alumni outside health sciences. To understand the micro-level experience of transitioning from Medicaid to private insurance, it is important to hear from a variety of students of a diverse, representative array of college majors. These findings will better inform their employers as to how to help them understand their new health benefits.

Interview subjects described self-medicating and avoiding the healthcare system to avoid paying copayments. This is a subject for future research. What are the health implications of avoiding the healthcare system for a patient population—former Medicaid patients—who bear a disproportionate health burden having spent some portion of their life in poverty? A related topic for future research and educational activities is to help new private insurance recipients understand their benefits. Based on the research interviews, it is unclear if the interview subjects were unaware that there is no cost sharing with preventative visits, and it is also unclear if interview subjects were aware of how private health insurance tiers copayments by physician specialty. The private sector has an opportunity here to innovate, improve outcomes, minimize inequities, and ensure resilience by better on-boarding their new hires.

Every interview subject described some degree of difficulty with health insurance transition with two individuals not knowing that they were disenrolled from Medicaid and had private health benefits until trying to use their Medicaid card and being denied care. For these interview subjects, they did not know how to read their paystubs and interpret the payroll reductions. One of the interview subjects did not know that they were enrolled in a healthcare savings account with additional money deducted from their paycheck. Future research is warranted to study the lack of understanding of payroll reductions as well as studies of educational campaigns that help with job transitions.

Finally, additional research is needed to understand the experience of paying for something that was in their experience previously free. Interview subjects described being on Medicaid for their entire lives, and though they were poor, they never had to worry about accessing the healthcare system or paying for care. Now that the interview subjects out-earn the Medicaid income restrictions, they are charged a premium, deductible, coinsurance, and/or copay for healthcare and are experiencing the stress of affording care. The stress of paying for services that were historically free made half of the interview subjects describe assuming that they would be on Medicaid again in the future, and they want to be sure to have access to affordable healthcare. Furthermore, interview subjects describe distrusting the medical system now that they have private insurance, responding that doctors love their copays and feeling like health insurance is a scam.

## Data Availability

The raw data supporting the conclusions of this article will be made available by the author, without undue reservation.
